# Do medium and Context Matter when learning from multiple complementary Digital texts and videos?

**DOI:** 10.1007/s11251-022-09591-8

**Published:** 2022-09-11

**Authors:** Lucia Mason, Christian Tarchi, Angelica Ronconi, Lucia Manzione, Natalia Latini, Ivar Bråten

**Affiliations:** 1 Department of Developmental Psychology and Socialisation, University of Padua, via Venezia 8, 35131 Padua, Italy; 2 Department of Education, Languages, Intercultures, Literatures, and Psychology, University of Florence, Florence, Italy; 3 Department of Education, University of Oslo, Oslo, Norway

**Keywords:** Digital texts and videos, Informational context, Behavioral engagement, Integrated understanding, Learning from complementary sources, Calibration of performance

## Abstract

Students more than ever learn from online sources, such as digital texts or videos. Little research has compared processes and outcomes across these two mediums. Using a between-participants experimental design, this study investigated whether medium (texts vs. videos) and context (less authoritative vs. more authoritative), independently and in concert, affected students’ engagement, integrated understanding, and calibration. The two mediums presented identical information on the topic of social media, which was distributed across two complementary texts in the text condition and across two complementary videos in the video condition. In the less authoritative context, the two information sources (texts or videos) were posted by a friend on Facebook; in the more authoritative context, the same information sources (texts or videos) were posted by a professor on Moodle. Results showed a main effect of medium on behavioral engagement in terms of processing time, as students used longer time watching the two videos than reading the two digital texts. No other main medium or context effects were statistically significant; nor were there any interaction effects of medium with context on any of the outcome variables. The findings are discussed in light of the alternative hypotheses that guided the study and the directions it suggests for future research.

**Supplementary Information:**

The online version contains supplementary material available at 10.1007/s11251-022-09591-8.

## Introduction

Digital materials have gained a prominent role in academic learning contexts. Although the COVID-19 pandemic, with its shift toward distance learning, undoubtedly has accelerated this development, digital learning materials were quite popular well before schools and universities closed down due to the public health emergency. Digital materials include texts and videos. Although digital texts have a longer history in educational contexts than have videos, the latter medium has gained immense popularity in the last decade. With the appearance of YouTube and other platforms for sharing videos, watching educational videos has become a common way of acquiring information (List, 2018).

Research comparing students’ comprehension of printed and digital informational texts has flourished in recent years, and several meta-analyses of the impact of reading medium are available (Clinton, 2019; Delgado et al., 2018; Kong et al., 2018). These meta-analyses consistently showed a screen inferiority effect, which means that text comprehension was poorer after reading on screen. When comparing studies before and after 2013, Kong et al. (2018) found a weakening of the negative impact of digital reading. In contrast, Delgado et al. (2018) found that the screen disadvantage increased over time (from 2000 to 2017), as well as when reading occurred in a fixed time frame and the texts were expository or a mix of expository and narrative. Clinton (2019) confirmed the superiority of reading on paper for the comprehension of expository texts, and also found that readers’ metacognitive judgments about their comprehension were better when reading on paper. A plausible explanation for the screen inferiority effect is the shallowing hypothesis (Annisette & Lafreniere, 2017). It states that when reading on a screen, students are inclined to process information more superficially than when reading in print because their usual interactions with digital devices are quick and in search for immediate rewards (e.g., likes, sharings, entertainment). Thus, students are prone to develop a mental habit that is not conducive to performing well on tasks requiring sustained attention, such as comprehending challenging single and, especially, multiple texts.

However, potential differences between digital informational texts and videos have hitherto received very limited attention (List, 2018; List & Ballenger, 2019, Salmerón et al., 2020). Because both digital texts and videos can be used to learn about unfamiliar, complex issues, it is highly relevant to understand not only how students comprehend information from a single text or a single video but also how they integrate information across multiple texts and multiple videos (List & Ballenger, 2019).

Further, the role of the informational context is an underfocused issue within cross-medium comprehension research. Yet, the informational context can be assumed to impact processes and outcomes of comprehension and metacomprehension (Britt et al., 2018). The current investigation focused on a crucial feature of the informational context, that is, its authoritativeness. Presumably, learners may decide to process information more deeply when it is accessed in a more authoritative informational context. In the present study, we operationalized authoritativeness in reference to the discursive context that elicited the need for more information on the topic: the person who posted the digital texts or the videos, and the platform on which the texts and the videos were shared. The overall purpose of our study was to explore potential effects of informational medium (texts vs. videos) and context (more authoritative vs. less authoritative) on university students’ behavioral engagement, integrated understanding, and judgment of performance.

### Integrated comprehension of complementary texts

The influential Construction-Integration (CI) model (Kintsch, 1998) posits that the process of text comprehension occurs at the levels of the surface structure, the textbase, and the situational model. At the surface level, the linguistic input, that is, the words, sentences, and the relations among them, are represented. At the textbase level, the micro- and macro-structures of the text are represented through inferencing that creates connections among different parts of the text. Finally, at the situation model level, a coherent, deeper comprehension of the text is achieved through the integration of textual information and relevant prior knowledge.

Whereas the CI model essentially concerns the comprehension of a single text, comprehension of complex issues often requires the reading of multiple texts. Accordingly, a range of studies have investigated the comprehension of multiple texts that present conflicting information on the same issue or phenomenon, which represents a very common situation when learners search for information on complex or controversial topics on the Internet. Much of this research has been framed by the Documents Model (DM), which was proposed by Perfetti et al. (1999) more than 20 years ago (for an updated version, see Britt & Rouet, 2012). This model extends the CI model by including two additional layers: the situations model or the integrated mental model and the intertext model. The first of these concerns the integration of content information across texts; the second concerns the integration of content information and source information (e.g., about the author) as well as relationships between the different sources (e.g., the authors of different texts). The intertext model allows readers to achieve overall coherence despite inconsistent or conflicting information, for example by realizing that different perspectives may be due to different levels of expertise or different motives on the part of the authors. Still, much research has documented that such integration of information across multiple conflicting texts is a major challenge for learners across educational levels (Barzilai et al., 2015, 2018; Mason et al., 2018; Salmerón et al., 2018).

In comparison, fewer studies have investigated learning from multiple complementary texts (for review, see Firetto, 2020). Such texts include distinct information that must be combined, rather than reconciled, to mentally represent a larger whole of textual content. That is, when dealing with multiple complementary texts, integrated understanding requires a combination of supplemental information across texts to create a more comprehensive representation than what can be derived from any single text (List & Alexander, 2018). In this way, complementary texts also differ from multiple texts that merely present overlapping information for corroborative purposes (Wiley et al., 2009).

In a recent experiment, Latini et al. (2019) examined undergraduates’ integration of information across complementary printed or digital texts on the topic of social media. Prior research indicating a disadvantage for screen-based reading with respect to comprehension performance (Clinton, 2019; Delgado et al., 2018) is consistent with the shallowing hypothesis (Annisette & Lafreniere, 2017). Latini et al. (2019) investigated whether reading purpose ‒ reading for pleasure versus in preparation for an exam ‒ moderated any effects of reading medium on behavioral engagement and text integration. As hypothesized, an interactive effect of reading medium with reading purpose emerged. Specifically, when students read digital or mixed texts for an exam, they displayed higher behavioral engagement (i.e., longer reading time) than when reading the same texts for pleasure, whereas reading purpose did not matter for their behavioral engagement when they read on paper. Using the length of post-reading written responses as an indicator of behavioral engagement, results showed that students reading printed texts displayed higher behavioral engagement when reading for an exam than when reading for pleasure, whereas no effects of reading purpose was observed when reading digital texts. Finally, Latini et al. found that behavioral engagement in the form of written response length fully mediated the effect of reading purpose on text integration when students read printed texts. In sum, this study indicated that the role of reading medium on student engagement and performance may be moderated by contextual factors. Accordingly, we maintained a focus on context in the current investigation.

### Integrated understanding of complementary texts and videos

Comparable comprehension processes are assumed to be involved in meaning making regardless of the modality, such as during both reading and listening (McNamara & Magliano, 2009), with the CI model by Kintsch (1998) guiding research across modalities. Thus, after recognizing words either through reading or listening, the processes involved in understanding words, sentences, and their connections to form a coherent mental representation seem to be independent of modality (McNamara & Magliano, 2009). Accordingly, the CI model (Kintsch, 1998) and the DM (Perfetti et al., 1999) can presumably be extended to understand learning with other mediums, such as videos, which have gained enormous popularity both in and out of the classroom (Baron, 2021).

Among the various types of videos that can be identified (e.g., Fiorella et al., 2019; Merkt et al., 2011), following Salmerón et al. (2020), we used “Internet videos” in the current study. In such videos, which are readily available on YouTube, the author typically speaks in front of a camera to present a point of view on a particular topic. Many of these videos are focused on educational content that pertains to different disciplines. As noted by Delgado et al. (2021), educational video blogs (vblogs) are very popular among young people and constitute a large share of the YouTube videos. Among the characteristics of such videos is interactivity, that is, viewers can pause, re-watch some parts, or advance fast. This makes videos comparable to texts where readers can re-read sentences and paragraphs as well as skip parts of the text. The interactivity of the learning material is a relevant characteristic for learning (Delgado et al., 2021); in fact, studies comparing digital texts and interactive videos have shown no medium difference with respect to comprehension (e.g., Burin et al., 2021; Delgado et al., 2021; Salmerón et al., 2020; Tarchi, 2021). For example, no effect of medium on comprehension performance emerged in a recent study that compared digital text, video in the form of audio explanation with written keywords, and video that also added dynamic decorative and irrelevant images as seductive details to the previous type (Burin et al., 2021).

Compared to digital texts, “Internet” videos add a representation of the information source as a visual entity. Further, such videos involve both the auditory and the visual channel. According to the theory of multimedia learning (Mayer, 2014), students learn better when information is presented through multiple mediums than through a single medium (e.g., a written text). Specifically, the multimedia principle is based on the assumption that two different channels ‒auditory-verbal and visual-pictorial ‒ process textual and visual information independently. When information is provided via both narration and images, students have the opportunity to use both channels for encoding and retrieval. However, for the multimedia principle to be effective, students need to process the information actively, that is, to engage in the crucial processes of selecting relevant information when encoding the narrated information, organizing the sequence of narrated and visual information, and integrating information across modalities and with prior knowledge (Mayer, 2014).

Given the popularity of both digital texts and videos, it is both theoretically and practically relevant to compare the comprehension and integration of information from complementary materials in the two mediums. To the best of our knowledge, only List and colleagues (List, 2018; Lee & List, 2018; List & Ballenger, 2019) have thus far conducted systematic research on how students process, comprehend, and integrate information from complementary digital texts and videos, respectively. List (2018) focused on the strategies used by undergraduates in processing expository texts and videos. Although no difference was found between those who read the two texts and those who watched the two videos with respect to the number of strategy categories identified in self-reports, the nature of the processing strategies varied by being both trans-symbolic and symbol-specific. Moreover, neither comprehension nor integration differed statistically significantly across the conditions (i.e., texts vs. videos; List, 2018).

In another study using the same materials, Lee and List (2018) focused on the processing of texts and videos as reflected in students’ actual annotations. Findings showed that participants annotated more often during text than during video processing, and the annotations they made reflected higher-level strategies (e.g., inferential questions or self-explanations) to a greater extent in the former condition. Finally, comprehension was found to be better in the video condition, whereas integration of information across the two information sources was better in the text condition.

Further, List and Ballenger (2019) had pre-service teachers read two complementary texts, one mainly expository and one mainly narrative, watch two corresponding videos, or read one text and watch one video. Data regarding the time spent reading/watching and processing strategies were collected. Results showed that participants reportedly used higher-level strategies to a greater extent when processing information presented in the texts than in the videos. They also spent a longer time processing the first, expository text compared to the first corresponding video, whereas no differences were found between the second, narrative text and the corresponding video. Finally, results showed that integration of information across sources was rather limited in all conditions (List & Ballenger, 2019).

Of note is also that Salmerón et al. (2020), in a recent study with primary school children, investigated the evaluation and integration of multiple texts and videos. However, the multimodal learning materials used in that study were not complementary as in List’s (2018; Lee & List, 2018; List & Ballenger, 2019) research. Findings showed that although the medium did not affect students’ memory for source information, students seemed to consider the information presented in the videos more believable than information presented in the texts. Nevertheless, students made more integrative inferences across texts than across videos.

In sum, the results of the cited studies on learning from multiple texts and videos are inconclusive with respect to comprehension performance. However, they suggest that videos are attended to for a shorter time than are texts and that integration of information is a challenging task, in particular, when younger students learn from videos. A possible disadvantage of videos may be explained in reference to the shallowing hypothesis (Annisette & Lafreniere, 2017). Most students use videos for entertainment. Even when they interact with videos for educational purposes, they may process them superficially, making it difficult to construct a coherent mental representation of complex informational content (Delgado et al., 2021; Salmerón et al., 2020).

Another issue related to the comprehension of texts and videos concerns the calibration of performance. This construct refers to the fit between metacognitive judgments and actual task performance (Alexander, 2013; Prinz et al., 2018). Calibration is important for appropriate decision making, which requires that students monitor and gauge their performance accurately (Rutherford, 2012). As such, calibration may influence decisions to continue studying learning materials and processing the content or to give up (Hacker et al., 2008). Calibration is typically measured by obtaining a judgment of performance prior to performance (i.e., a prediction) or a judgment after performance (i.e., a postdiction). When the contrast between self-judged performance and actual performance is small, a student is considered well calibrated; conversely, when the contrast is large, the student is considered poorly calibrated. Both over-confidence and under-confidence thus indicate poor calibration. Research has indicated that students typically tend to be over-confident about their level of comprehension, which has implications for executive control and regulatory strategies (Hacker et al., 2008).

With respect to printed and digital text comprehension, in particular, a meta-analytic study by Clinton (2019) showed that students may be more inaccurate in self-evaluating their performance when reading on screen as compared to on paper, which also seems consistent with the shallowing hypothesis. Likewise, research on the comprehension of video-recorded lectures in online learning contexts has shown that students are typically not well calibrated, although their self-evaluation can become more accurate by interpolating video presentation with testing (Szpunar et al., 2014). To the best of our knowledge, only one study has focused on calibration of comprehension performance when comparing (single) texts and videos (Tarchi et al., 2021). In that study, no differences in students’ self-evaluation were found.

In addition to our focus on engagement, integration, and calibration when learning with digital texts and videos, we set out to explore the potential influence of the context in which the digital texts and videos were presented. Thus far, no research has investigated this particular combination of medium and context. As such, our study uniquely extends prior research in this area.

### Informational context, engagement, and integration

The reading as problem solving (RESOLV) model by Britt and colleagues (Britt et al., 2018; Rouet, Britt, et al., 2017) is a useful tool for understanding the potential role of context in reading. Basically, RESOLV posits that readers initially construct a mental model of the context in which reading takes place. This mental representation includes aspects of the context perceived as relevant by the readers and the inferences they make about the context. Based on their context model, readers also build a task model, which is a representation of the end goal and the means to pursue it. For example, the representation of the context may include the reading instruction in the form of a specific rquest, but it may also include information about the requester and her or his relationship with the reader (e.g., a professor or a friend). The construction of a context model is based on readers’ attention to, selection, and processing of context (physical and social) cues that they perceive as important. As such, it is a subjective representation derived from cues in the environment as well as readers’ inferences or elaborations (Rouet, Britt, et al., 2017).

In the current study, we focused on one particular aspect of the informational context that students might include in their context model: its authoritativeness. The requester (e.g., teacher, peer) and the available information resource (e.g., type of digital venue) are among the essential characteristics that define authoritativeness and potentially have a direct impact on reading processes and outcomes (Rouet, Britt, et al., 2017). A more authoritative informational context creates higher expectations about information accuracy and credibility. Specifically, a more authoritative context may be a context in which a professor, after a classroom discussion, recommends and publishes documents on Moodle, which is a platform for the management of university teaching, so that the students may learn more about the discussed topic. In contrast, a less authoritative context may be a context in which a friend, after a discussion with other friends out of school, recommends and publishes documents on Facebook. In this regard, studies on the use of Facebook for learning purpose have revealed issues regarding, for example, managing and synthesizing information and poor time management (Niu, 2019).

The authoritativeness of the informational context may, accordingly, moderate both processes and outcomes of reading multiple complementary texts or watching multiple complementary videos. Regarding processes, longer time may be devoted to the processing of materials deemed trustworthy as compared to untrustworthy. Higher behavioral engagement may thus occur when interacting with learning materials in a more authoritative informational context and lower behavioral engagement may occur when interacting with learning materials in a less authoritative context. Distinct from other types of engagement, such as cognitive, emotional, and agentic engagement (Sinatra et al., 2015), behavioral engagement concerns active, observable involvement in a learning activity or task. In other words, behavioral engagement reflects students’ attentiveness, persistence, and investment of time (Ben-Eliyahu et al., 2018; Wu & Wu, 2014). Engagement is considered to be supported by both cognition (e.g., background knowledge) and motivation (e.g., intrinsic motivation) (Guthrie & Klauda, 2016). In previous pertinent research, two valid, objective measures of behavioral engagement have been used. The first one is the time spent processing a given material, for example reading a printed or digital text. Processing time has been found to predict multiple text comprehension (e.g., Bråten et al., 2014; List et al., 2019). A second, validated measure of behavioral engagement is the extent of the written task product used to assess comprehension performance in response to open-ended integrative questions or essay tasks (Bråten et al., 2022; Latini et al. 2019). Bråten et al. (2022) investigated the effects of two components of behavioral engagement, writing time and response length, on text comprehension. Findings showed that these components had unique and differential effects on comprehension and that behavioral engagement mediated the effects of cognitive and motivational individual differences on comprehension performance.

In sum, it seems relevant to explore whether the authoritativeness of the informational context might moderate students’ behavioral engagement with the digital text and video materials, their integrated understanding of content information, and their calibration of the performance. When complementary texts or videos are presented in a more authoritative informational context, they may receive more attention and, consequently, lead to better comprehension performance than when texts and videos are presented in a less authoritative context, given that authoritativeness is mentally represented as a relevant aspect in the context model (Rouet, Britt, et al., 2017).

## Research questions and hypotheses

This study is a unique contribution by investigating potential effects of both medium and context, as well as their interaction, on behavioral engagement, integration of information, and calibration. Regarding the medium factor, we compared complementary digital texts and videos on the topic of social media. Regarding context, we compared a more authoritative context in which digital texts/videos were posted by a professor on the course page on Moodle after a classroom discussion with a less authoritative context in which the texts or videos were posted on Facebook by a friend after a discussion out of school. We also considered it relevant to include two mixed conditions combining texts and videos in the research design because students very often interact with both texts and videos to learn about an unfamiliar topic. These conditions could therefore be considered to complement the design and reflect the learning materials used by today’s students.

Three research questions (RQs) guided our study:

RQ1: Does medium (digital text vs. video) affect students’ behavioral engagement as reflected in the time spent processing complementary source materials and in the length of their written responses to the post-reading comprehension assessment, and does informational context (more authoritative vs. less authoritative) moderate their behavioral engagement?

RQ2: Does medium (digital text vs. video) affect students’ integration of information across digital texts and videos, respectively, and does informational context (more authoritative vs. less authoritative) moderate their integrated understanding?

RQ3: Does medium (digital text vs. video) affect students’ calibration of performance, and does informational context (more authoritative vs. less authoritative) moderate their calibration?

For both RQ1 and RQ2, we formulated alternative hypotheses considering the thin research base on these issues and the mixed results obtained in previous studies. For RQ1, based on available results on the processing of texts and videos (List & Ballenger, 2019) and the shallowing hypothesis (Annisette & Lafreniere, 2017; Salmerón et al., 2020), one might expect an effect of medium because students could be more engaged (i.e., use more time and produce longer written responses) when working with the digital texts. Also, based on preliminary prior work on the effects of context models (Rouet, Rupp, et al., 2017), one might expect that the authoritativeness of the informational context could moderate behavioral engagement, especially when working with the video materials that may elicit more superficial processing. Thus, any differences in the two indices of behavioral engagement in the favor of texts might be reduced or even eliminated when students watch videos in a more authoritative context. This is because participants watching videos posted by a professor on the Moodle course page after a classroom discussion could interact more deeply with the content compared to participants watching videos posted by a friend on Facebook after a discussion out of school.

However, one might also entertain the possibility that the medium would not matter with respect to behavioral engagement, at least not with respect to processing time (List & Ballenger, 2019). It is also a possibility that the context would not moderate the effect of medium on behavioral engagement because authoritativeness might not be represented in participants’ context model and, thus, not drive students’ further engagement with the task (Rouet, Britt, et al., 2017). This would be consistent with prior research indicating that students more often than not disregard source characteristics and pay attention only to the content of the learning materials (Bråten, Stadtler, et al., 2018).

For RQ2, based on the results of the few studies on content integration across texts or videos at different educational levels (Lee & List, 2018; Salmerón et al., 2020), one might expect a main effect of medium in favor of text. Also, one might expect an interactive effect of medium with context on integrated understanding, specifically that context would matter more for video watchers. Previous studies have suggested that students may pay more attention to content when watching videos in a more authoritative informational context (Kammerer et al., 2016; Salmerón et al., 2020), and that such engagement may translate into deeper processing and, in turn, better integration of information (Bråten, Brante, et al., 2018; Latini et al., 2019; List & Alexander, 2018). Videos watchers in a more authoritative context might therefore outperform videos watchers in a less authoritative context.

However, based on existing studies (Lee & List, 2018), one might also expect that there would be no difference between mediums with respect to content integration. Also, students might not represent the key feature of authoritativeness in their context model (Rouet, Britt, et al., 2017) and, therefore, not improve their comprehension performance in the more authoritative context.

For RQ3, we opted for a purely exploratory approach, given that only one study in this area addressed calibration of comprehension performance, yet compared reading a single printed text with watching a single video (Tarchi et al., 2021). That study did not show any reliable difference in terms of students’ metacognitive judgment about their performance as a function of medium. In her meta-analysis, Clinton (2019) reported better calibration of comprehension performance associated with reading on paper than on screen, but that analysis compared print and digital reading and did not include video watching.

Of note is that we included the two mixed conditions of text and video in a more or less authoritative informational context for the reasons previously mentioned. However, we were not able to ground this in prior theory and research specific hypotheses about the comparative effects of learning materials presented in both textual and video mediums. We did not integrate, therefore, the two mixed conditions in our alternative hypotheses.

Testing for effects of medium and context cannot ignore the potential role of individual differences, both cognitive and motivational, that may be associated with behavioral engagement, integration, and calibration. In the current study, we took several individual differences into account, including perceived prior knowledge, reading comprehension, cognitive reflection, and task value (i.e., the perceived value of learning from texts and videos, respectively). Perceived prior knowledge may influence processing, comprehension of informational materials, and calibration of comprehension performance. For example, those who perceive themselves as knowledgeable may perceive the task as easy and approach it in a way that has negative consequences for learning processes and outcomes (Tarchi et al., 2021). Reading comprehension is foundational to both single- and multiple-text comprehension (Mason et al., 2020) and therefore needs to be partialled out when studying potential effects of medium and context on processing, integration of information, and calibration. It should be noted that we considered it pertinent to include reading comprehension as a covariate even though participants in two conditions did not read texts but only watched videos. However, these videos were spoken versions of the written texts (see the Learning materials section), and in the literature, there is evidence that adults’ oral language comprehension and written text comprehension are related, at least in transparent languages (Goncalves et al., 2021; Tobia & Bonifacci, 2015).

Cognitive reflection, which refers to a disposition toward rational thinking, likely sustains task engagement and the construction of high-quality mental representations of content regardless of medium (Frederick, 2005). Finally, the motivational variable of task value, that is, the importance and usefulness attributed to a task, may have an impact on processes and outcomes of reading or watching informational materials (Tarchi et al., 2021). In sum, controlling for these variables may ensure that any effects due to our manipulation of the two independent variables, medium and context, were independent of perceived prior knowledge, reading comprehension, cognitive reflection, and motivation to learn from texts and videos.

## Method

### Participants

Participants were 255 Italian students in higher education. Their mean age was 24.03 years (*SD* = 5.98) and 83.9% were female. Most (71.4%) were bachelor students enrolled in programs of primary school teacher education (40.4%) and psychology (19.2%). Other less represented fields of study were political science, engineering, medicine, and pharmacy. Italian was the first language for almost all (98%) participants, and non-native speakers were competent in Italian. Participants reportedly preferred learning from videos (*M* = 6.79, *SD* = 2.16) more than learning from digital texts (*M* = 4.31, *SD* = 2.08), *t*(154) = 12.01, *p* < .001, Cohen’s *d* = 0.75). The study was approved by the ethics committee at the university of the first author. The majority of the participants were recruited during regular lectures and volunteered to participate for course credit. Our sample size was justified by an a priori power analysis performed in G*power (Faul et al., 2007), based on α = 0.05, 1 – β = 0.95, and an estimated medium effect size (*f* = 0.25).

### Experimental conditions

To address our hypotheses, we manipulated the two independent variables of medium and context. As already described, medium was either two digital texts or two videos. The context was either more authoritative or less authoritative. As previously introduced, authoritativeness was operationalized by the discursive context that elicited the need for more information on the topic, the person who posted the learning materials, and the digital venue or platform on which the materials were posted. In the less authoritative context, the materials were posted by a friend on Facebook after a discussion with other friends out of school. In the more authoritative context, they were posted by a professor on Moodle after a classroom discussion.

This context manipulation did not occur in a naturalistic situation, that is, in an authentic classroom where students really discussed the examined issues. Thus, only semi-authenticity characterized our manipulation. However, in learning research students are usually asked “to imagine” a situation (Latini et al., 2019; McCrudden et al., 2010).

Of note is that the content of the texts and videos and source information were exactly the same across contexts. As previously mentioned, we also included two mixed conditions. These were reading a text and watching a video on Facebook, and reading a text and watching a video on Moodle. Thus, participants were randomly assigned to one of six conditions: (1) two texts on Facebook; (2) two texts on Moodle; (3) two videos on Facebook; (4) two videos on Moodle; (5) one text and one video on Facebook; (6) one text and one video on Moodle. In the mixed conditions, the order of the two mediums was counterbalanced.

### Materials

**Learning materials**. Depending on the experimental condition, participants read two digital expository texts or watched two videos on the topic of social media with the same content as the texts used by Latini et al. (2019, pp. 11–12), which were translated into Italian for the purpose of this study. The texts had mainly been adapted from bachelor level textbooks and popular science articles (see Supplementary materials). One text/video was titled “Social Media ‒ Friend or Foe?” and the other “Social Media = Social People?”. The two texts/videos presented complementary information about four issues related to social media: (1) psychological aspects of social media use, (2) educational level in relation to social media use, (3) the possible impact of social media use on friendships, and (4) gender in relation to social media use. For instance, concerning the possible impact of social media use on friendships, one text/video described that making new friends is much easier on social media, while the other, complementary text/video described that the quality of friendships may differ between social media and real life. Combination of information about each of the four aspects across the two texts/videos was therefore required to get a complete, integrated understanding of each issue.

At the beginning of each text/video, information about the source was presented in terms of publication, author’s name and occupation, and date of publication. Specifically, regardless of the medium and context, the materials were presented as taken from the online version of a well-known and prestigious Italian newspaper (*Il corriere della sera*) and authored by two different female journalists. That is, one text/video was attributed to one of these journalists, while the other text/video was attributed to the other one. For both journalists, fictitious names were used. Of note is that the content of the texts/videos and the source information were held constant as they were exactly the same across contexts. This allowed us to compare contexts in which the same information content and the same source information were included. The learning materials were feature articles, not news articles, and had mainly been adapted from bachelor level textbooks and popular science articles (the texts are included in the online Supplementary materials).

The length of the two written/spoken Italian texts was exactly the same: 771 words. As an indication of text difficulty, we used the only tool available for Italian texts, the Gulpease index (maximum readability = 100). The readability score was 53 for one text and 48 for the other, indicating that they were at the level of readers with a high school diploma and that some effort, therefore, was required by the participants to gain a good understanding of the content.

Videos were “Internet videos” typically found on YouTube, in which the author speaks in front of a camera to give a presentation on a particular topic. No subtitles, graphics, animations, or seductive details were included, reducing extraneous cognitive load to a minimum. Participants could control the videos by stopping and re-watching them whenever they wanted.

The two female journalists who spoke on the two videos were similar in physical appearance (e.g., about the same age, both had short black hair and wore glasses) and were dressed similarly (both wore a white sweater). They also used a similar tone of voice. The two videos were prepared by transcribing the two written texts and having the journalists present them orally in the videos. Of note is that the two speakers read the texts that were in front of them, although not visible. Thus, they did not speak as they would typically speak to an audience on a topic, which would require longer time. While reading, they spoke as naturally as possible but faster than in typical speech. The reason for this presentation was that we wanted to make the processing times for the texts and the videos comparable.

**Fig. 1:**
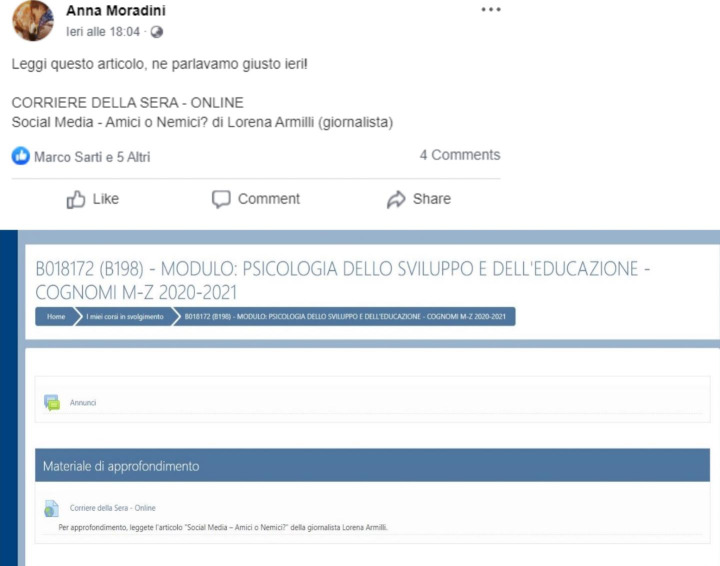
Mock Ups for the Unauthoritative and Authoritative Contexts with Textual Materials “Social Media: Friend or Foe?”

**Fig. 2:**
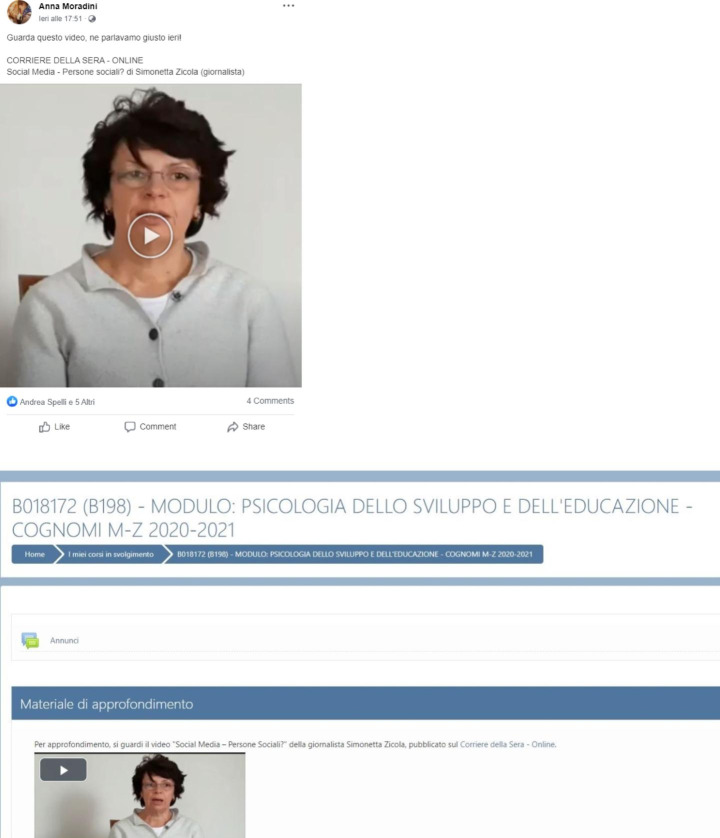
Mock Ups for the Unauthoritative and Authoritative Contexts with Video Materials “Social Media = Social People?”

The video “Social Media ‒ Friend or Foe?” lasted 5 min and the video “Social media = social people?” lasted 5 min and 10 s. For contextualization, the complementary texts/videos were embedded in the two online platforms (i.e., Facebook and Moodle) in authentic ways, as shown in Figs. 1 and 2, respectively.

### Dependent measures

**Processing time**. The first measure of behavioral engagement was the time used for reading the first and the second text, watching the first and the second video, or reading a text and watching a video.

Processing time was registered by the online system. In the statistical analyses, we used the total processing time for the learning materials.

**Response length**. The second measure of behavioral engagement was the length of the responses on the written comprehension assessment. It was computed by counting the number of words in the written responses to the four questions. In the statistical analyses, we used the total response length as an index of effort.

**Information integration**. Following Latini et al. (2019, p. 5), we assessed integrated understanding in all conditions by asking participants to answer four short essay questions in writing, one regarding each of the issues related to social media that were discussed in the learning materials. We opted for this kind of questions rather than intertextual verification tasks in order to assess the integrated mental model of the Documents Model framework (Britt & Rouet, 2012). Compared to receptive verification tasks, essay writing is an expressive task that requires an active integration of content across texts (Primor & Katzir, 2018). According to Barzilai et al. (2018), such essay tasks are the most frequent approach to assessing intertextual integration across multiple informational texts (i.e., the integrated mental model).

Scoring was based on whether participants correctly represented the information about the four issues that were discussed in the learning materials and whether they integrated information across texts/videos. Specifically, responses were awarded 0–3 points depending on their degree of integration: 0 points were given to irrelevant, incorrect, or no responses; 1 point was given to correct responses describing an issue related to social media as discussed in only one of the texts/videos; 2 points were given to correct responses describing an issue as discussed in both texts/videos without any integration across texts/videos; and 3 points were given to correct responses describing an issue as discussed in both texts/videos and integrating information about that issue across texts/videos. Specifically, integration was coded based on the use of causal and adversative connective words in the written responses. Examples of causal connectives are *hence*, *therefore*, *because*, *consequently*, and *since*, with the use of such words indicating a combination of information across texts/videos to provide a more complete answer to the question. Examples of adversative connectives are *however*, *whereas*, *on the other hand*, and *in contrast*, which indicate a combination of information across texts/videos through comparing and contrasting to provide a more complete answer (Halliday & Hasan, 1976; Latini et al., 2019). The maximum score was 12 and the total score for the four questions was used in statistical analyses. Examples of questions and answers are provided in Table 1 S of the supplementary materials.

The third and fourth authors scored the responses, blind to experimental condition. First, 30% of the responses were scored collaboratively in the presence of the first author. Then, all remaining responses were scored independently by the two authors, resulting in high interrater reliability coefficients (Pearson’s *r*) that ranged from 0.89 to 0.98 for the four questions. All disagreements were solved through discussion in the presence of the first author.

**Calibration of performance**. We retrospectively asked participants to self-evaluate how well they had responded to each of the four questions by using a 10-point scale (1 = not well at all, 10 = very well, maximum score = 40). Each question was used as a prompt for this judgment. We first computed a composite score and then a proportion score based on the relationship between the postdiction judgment and the maximum total score of 40. Further, we computed a proportion score for integrated understanding based on the relationship between the actual performance score and the maximum total score of 12. Finally, we subtracted the proportion score for integrated understanding from the proportion score for metacognitive judgment as a measure of calibration error or bias (Hacker et al., 2000).

### Control variables

**Reading comprehension**. Reading comprehension was measured using an Italian test battery for university students and young adults (Montesano et al., 2020). Participants read an expository text and answered 14 questions (maximum score = 14). The internal consistency reliability (McDonald’s ω) for participants’ scores on this test was 0.66.

**Perceived prior knowledge**. A six-item measure developed by Latini et al. (2019) was used as a proxy for participants’ prior knowledge about social media. Perceived knowledge (“I have knowledge about …”) has been shown to be a quite good indicator of scores on an actual knowledge task (Stanovich & West, 2008). The six items concerned knowledge about (a) the storage and use of personal information by social media, (b) similarities and differences among social media, (c) pros and cons of social media use, (d) different types of social media and their users, (e) social media as providers of news, and (f) how social media are used for marketing purposes. Items were rated on a 10-point scale (1 = disagree completely; 10 = agree completely). Internal consistency reliability (McDonald’s ω) for participants’ scores was 0.88.

**Cognitive reflection**. Cognitive reflection was measured with an Italian 6-item Cognitive Reflection Test that has been validated with Italian university students (Primi et al., 2015). Primi et al. added three new items to the original test by Frederick (2005). An example of the original items is: “If it takes 5 machines 5 min to make 5 widgets, how long would it take 100 machines to make 100 widgets?” (intuitive answer = 100; correct answer = 5). An example of an added item is: “Jerry received both the 15th highest and the 15th lowest mark in the class. How many students are there in the class?” (intuitive answer = 30; correct answer = 29). This test assesses cognitive reflection as the questions have an immediate but incorrect response that must be overridden by further reflection. Thus, the correct answer requires deliberation and rational (rather than intuitive) thinking (Toplak et al., 2014). McDonald’s ω for participants’ scores was 0.76.

**Task value**. Task value was assessed using two 11-item self-report inventories, one concerning the perceived value of learning from texts and one concerning the perceived value of learning from videos, which were adapted from Bråten et al. (2013). Each item was rated on a 10-point scale (1 = not at all; 10 = a lot). McDonald’s ω for participants’ scores was 0.79 for learning from texts and 0.76 for learning from videos.

### Procedure

Data were collected online through the Qualtrics platform. First, participants read and signed an informed consent form that also contained information about the estimated length of the session (approx. 50 min). Second, they completed a brief demographic survey. Third, they completed the tasks used to measure the control variables of task value, perceived prior knowledge, cognitive reflection, and reading comprehension. Fourth, participants were presented with a general instruction in accordance with their assigned experimental condition. This instruction informed that they should read two texts, watch two videos, or read a text and watch a video on Facebook or Moodle. They were encouraged to pay attention when reading/watching the texts/videos because they would be asked some questions about the content afterwards. They were also informed that they could re-read/re-watch the texts/videos as much as they wanted but could not go back after having clicked on “next” to continue to the second text/video. Fifth, before reading/watching the texts/videos, participants read a more specific instruction. Those assigned to the less authoritative context read the following: “You were involved in a lively discussion out of school with your friends about the use of social media. Today, one of your friends tagged you in two posts on Facebook that concern this topic and recommended that you read/watch them in order to continue the discussion with new information.” Participants assigned to the more authoritative context read the following: “You were involved in a lively classroom discussion about the use of social media during a lecture. Today, the professor recommended that you read/watch two texts/videos she posted on Moodle in order to continue the discussion with new information.” Depending on the experimental condition, participants read/watched the two texts/videos in a mock Facebook or Moodle environment that were similar to the two online platforms (see Figs. 1 and 2). After this specific instruction, participants started reading/watching the first text/video. The order of the texts/videos was counterbalanced in all conditions. Participants were not given a maximum time for processing the texts/videos. Sixth, after finishing reading/watching the texts/videos, participants in all experimental conditions read the following instruction: “You are now asked to answer four questions as clearly and completely as possible. Please do not respond only “yes” or “no” but write an argument on the basis of what you have read in the texts or heard in the videos. Answer all questions even if you are not sure.” Participants could not re-access the texts/videos while answering the questions. Finally, the last task asked participants to self-evaluate their performance when responding to each of the four questions.

## Results

We present the results organized by the research questions but start with some preliminary analyses.

### Preliminary analyses

Data were first tested for outliers and normal distribution. An outlier was defined as a value that deviates more than 2.5 standard deviations from the mean. Seven outliers were identified for one variable: the time spent processing the two videos. These outliers were removed for the analysis including this dependent variable. Table 1 reports the descriptive statistics and zero-order correlations for the entire sample. The measured variables were approximately normally distributed and, thus, suitable for parametric statistical analyses. Correlations showed that perceived prior knowledge was not related to any dependent variable. However, reading comprehension correlated positively with processing time for the learning materials (*r* = .20, *p* < .01), response length (*r* = .26, *p* < .01), and integration of information (*r* = .27, *p* < .01), and negatively with calibration error (*r* = − .18, *p* < .01). Cognitive reflection was positively associated with only response length (*r* = .15, *p* < .05) and integration of information (*r* = .17, *p* < .01). Likewise, task value of learning from texts correlated positively with response length (*r* = .22, *p* < .01) and integration of information (*r* = .25, *p* < .01), as well as negatively with calibration error (*r* = − .16, *p* < .05). Finally, task value of learning from videos correlated positively with processing time (*r* = .16, *p* < .05), response length (*r* = .16, *p* < .05), and integration of information (*r* = .17, *p* < .01).

Interestingly, integration of information was correlated positively with both processing time (*r* = .46, *p* < .01) and response length (*r* = .63, *p* < .01), and negatively with calibration error (*r* = − .81, *p* < .01). This indicates that, overall, participants who invested more time and effort in the task were also more likely to gain more integrated understanding and be better calibrated.

**Table 1:** Descriptive Statistics and Zero-Order Correlations for the Measured Variables

Variable	1	2	3	4	5	6	7	8	9
1. Perceived prior knowledge	-								
2. Reading comprehension	0.04	-							
3. Cognitive reflection	0.08	0.24**	-						
4. Task value for texts	0.07	0.25**	0.19**	-					
5. Task value for videos	0.15*	0.14*	0.08	0.58**	-				
6. Processing time^+^	-0.06	0.20**	0.04	0.07	0.16*	-			
7. Response length	-0.15	0.26**	0.15*	0.22**	0.16*	0.37**	-		
8. Integration of information	-0.56	0.27**	0.17**	0.25**	0.17**	0.43**	0.63**	-	
9. Calibration of performance	0.06	-0.18**	-0.12	-0.16*	-0.06	-0.30**	-0.48**	-0.81**	-
*M* *SD* Skewness	39.97 10.41 -0.49	10.22 2.49 -0.85	3.47 1.90 -0.27	90.47 11.37 -0.46	88.31 10.89 -0.56	671.14+ 383.50 0.64	192.96 109.56 0.94	6.32 2.95 -0.57	18.09 23.19 0.44

* *p* < .05; ** *p* < .01; + in seconds

Descriptive information for the individual difference measures by experimental condition is reported in Table 2S of the supplementary materials. A multivariate analysis of variance (MANOVA) did not indicate any statistically significant differences between the experimental conditions with respect to individual differences, *F*(25, 911.63) = 1.15, *p* = .269, η^*2*^_*p*_ = 0.023. Also, follow-up univariate tests showed that no single individual difference variable differed as a function of condition (see Table 2S). Individual differences were included as covariates in the statistical analyses that addressed our research questions to remove variance in the dependent variables associated with them (Field, 2018). Accordingly, we included the individual differences that correlated statistically significantly with the dependent variables as covariates in subsequent statistical analyses.

To address our research questions, we ran a 3 × 2 between-participants analysis of covariance (ANCOVA) for each of the four dependent variables: processing time, response length, integration, and calibration of comprehension performance. In these analyses, medium (texts, videos, text and video) and context (Facebook, Moodle) were the independent variables. We first tested the assumption of homogeneity of the regression slopes and found that this assumption was met for all analyses.

### Research question 1: Effects of medium and context on behavioral engagement

**Fig. 3:**
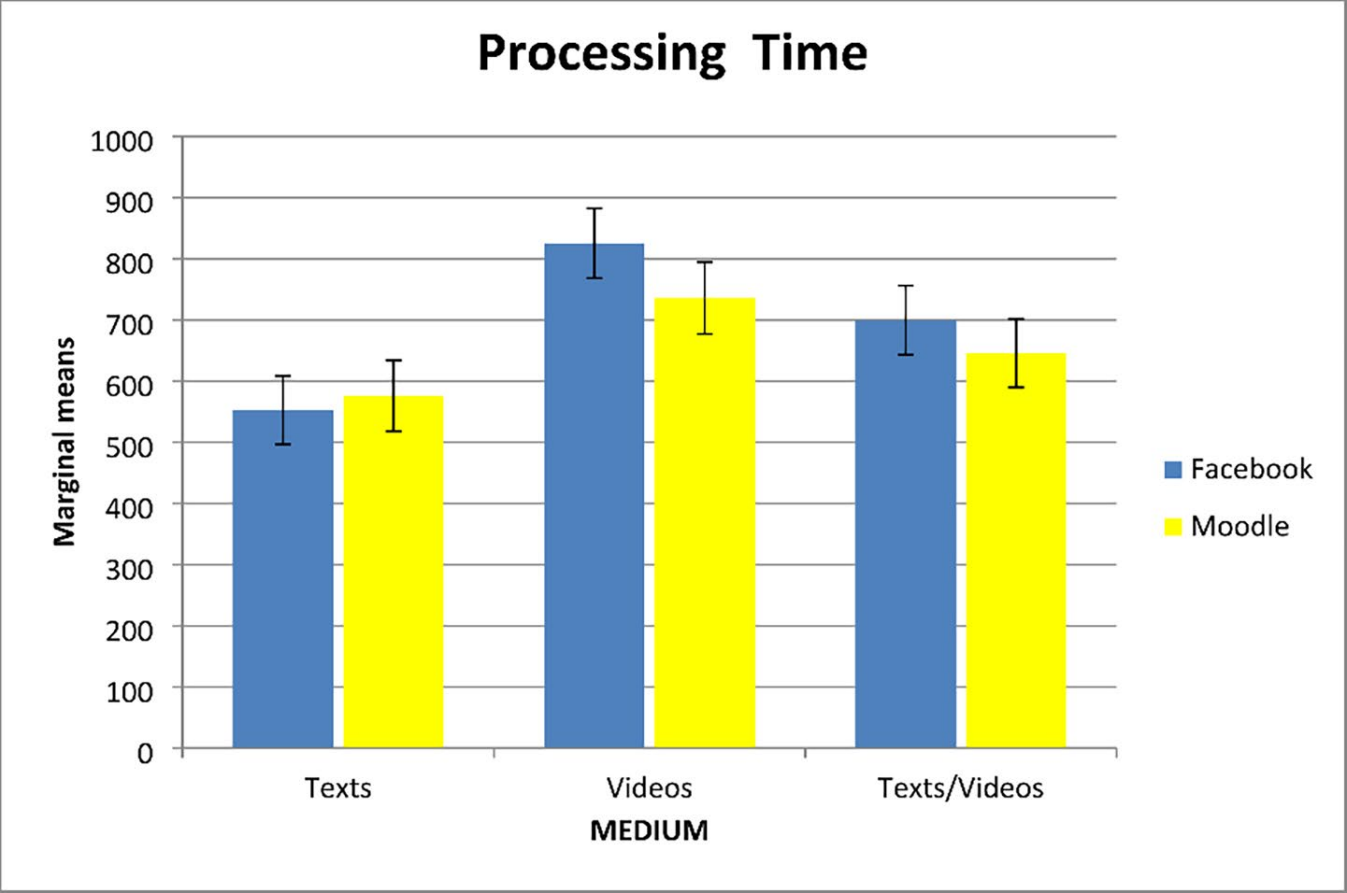
Processing Time as a Function of Medium and Context

In the first ANCOVA, processing time was used as a dependent variable indicating behavioral engagement and reading comprehension and task value for learning from videos were included as covariates. Results showed a main effect of medium^1^, *F*(2, 240) = 7.10, *p* < .001, η^*2*^_*p*_ = 0.056. Participants in the video condition spent longer time processing the information than did those in the text condition (*p* = .001, *d* = 0.52), while their processing time was not statistically significantly different from that of the mixed condition. Neither the effect of context, *F*(1, 240) = 0.74, *p* = .388, η^*2*^_*p*_ = 0.003, nor the interaction of medium with context, *F*(2, 240) = 0.51, *p* = .601, η^*2*^_*p*_ = 0.004, were statistically significant (see Fig. 3). Both the covariates of reading comprehension, *F*(1, 240) = 9.05, *p* = .003, η^*2*^_*p*_ = 0.036, and task value of learning from videos, *F*(1, 240) = 5.95, *p* = .015, η^*2*^_*p*_ = 0.024, uniquely adjusted processing time. These results indicate that better comprehenders and participants who highly valued learning from videos were more likely to invest time in processing the learning materials than were poorer comprehenders and participants who placed less value on learning from videos.

The second ANCOVA used response length as a dependent variable indicating behavioral engagement and included the covariates of reading comprehension, cognitive reflection, task value of learning from texts, and task value of learning from videos. This analysis showed no statistically significant main effect of medium, *F*(2, 245) = 0.01, *p* = .988, η^*2*^_*p*_ = 0.001, or context, *F*(1, 245) = 0.05, *p* = .821, η^*2*^_*p*_ = 0.001; nor was the effect of their interaction statistically significant, *F*(2, 245) = 0.43, *p* = .650, η^*2*^_*p*_ = 0.004. The covariate of reading comprehension uniquely adjusted response length, *F*(1, 245) = 9.99, *p* = .002, η^*2*^_*p*_ = 0.039, indicating that better comprehenders were more likely to write longer responses. Table 2 reports the estimated marginal means and standards errors for all dependent variables by medium and context.

**Table 2:** Marginal Means and (Standard Errors) for all Dependent Variables by Condition

	Unauthoritative context (Facebook)			Authoritative context (Moodle)		
	Texts (*n* = 43)	Videos (*n* = 43)	Texts/videos (*n* = 44)	Texts (*n* = 42)	Videos (*n* = 39)	Texts/videos (*n* = 44)
Processing time^+^	552.73 (55.76)	825.11 (57.09)	699.29 (56.68)	575.94 (57.73)	735.45 (58.62)	645.45 (55.76)
Response length	197.87 (16.25)	199.89 (16.13)	185.24 (16.03)	190.66 (16.31)	184.20 (17.05)	199.04 (16.08)
Integration	6.03 (0.43)	6.71 (0.42)	6.35 (0.42)	6.22 (0.45)	6.17 (0.45)	6.40 (0.42)
Calibration	17.70 (3.51)	15.44 (3.49)	20.01 (3.46)	18.37 (3.53)	17.05 (3.68)	19.80 (3.46)

+ In seconds

Overall, these findings showed that participants’ behavioral engagement ‒ as reflected in processing time and response length ‒ was essentially similar regardless of the medium, context, and their interaction. Further, the cognitive factor of reading comprehension and the motivational factor of valuing learning from videos seemed to play a role in students’ behavioral engagement.

### Research question 2: Effects of medium and context on integration of information

An ANCOVA with integrated understanding as the dependent variable and reading comprehension, cognitive reflection, task value of learning from texts, and task value of learning from videos as covariates showed no statistically significant main effect of medium, *F*(2, 245) = 0.29, *p* = .746, η^*2*^_*p*_ = 0.002, or context, *F*(1, 245) = 0.08, *p* = .777, η^*2*^_*p*_ = 0.001, and no statistically significant effect of their interaction, *F*(2, 245) = 0.38, *p* < = 0.684, η^*2*^_*p*_ = 0.03. The covariates of reading comprehension, *F*(1, 245) = 10.07, *p* = .002, η^*2*^_*p*_ = 0.039, and task value of learning from texts, *F*(1, 245) = 7.76, *p* = .030, η^*2*^_*p*_ = 0.020, uniquely adjusted integrated understanding, meaning that better comprehenders and participants who more highly valued learning from texts were more likely to integrate information across the learning materials than were poorer comprehenders and participants who placed less value on learning from texts. However, participants’ integrated understanding did not differ due to the medium, the context, or their interaction. Again, both cognitive and motivational factors explained variance in the outcome measure.

### Research question 3: Effects of medium and context on calibration

Finally, ANCOVA with calibration as the dependent variable and reading comprehension and task value of learning from texts as covariates, showed no statistically significant main effect of medium, *F*(2, 247) = 0.54, *p* = .582, η^*2*^_*p*_ = 0.004, or context, *F*(1, 247) = 0.05, *p* = .812, η^*2*^_*p*_ = 0.001; nor was their interaction statistically significant, *F*(2, 247) = 0.03, *p* = .968, η^*2*^_*p*_ = 0.01. The covariates of reading comprehension uniquely adjusted calibration, *F*(1, 247) = 5.86, *p* = .016, η^*2*^_*p*_ = 0.023, meaning that better comprehenders were more likely to display good calibration than were poorer comprehenders. These findings showed that metacomprehension as reflected in calibration of comprehension performance also did not differ by medium, context, or their interaction. Regardless of the medium, participants tended to overestimate their performance. The cognitive factor of reading comprehension contributed uniquely to the correspondence between participants’ metacognitive judgments about their performance and their actual performance.

### Confirmation of equivalence across conditions

Because the ANCOVAs showed that medium and context did not explain a statistically significant portion of the variance in any of the dependent variables, except for a moderate effect on processing time, we proceeded to confirm the equivalence across conditions through a set of Bayesian independent samples *t*-tests. For non-significant results, such tests allow for the quantification of the evidence for the null-hypothesis. Specifically, a Bayesian approach to hypothesis testing is comparative in nature (Jarosz & Wiley, 2014) and, thus, permits comparison of the amount of evidence supporting the null model (H0) with the amount of evidence supporting the alternative model (H1) by examining the Jeffreys-Zellner-Siow Bayes factor (BF; Wagenmakers et al., 2018). We calculated the BF_10_, which corresponds to the ratio between the likelihood of the data given H_1_ and the likelihood of the data given H_0_. The strength of the evidence supporting H_0_ or H_1_ was estimated adopting the criteria suggested by Raftery (1995) and Wetzels et al. (2011).

The analysis concerning an effect of the medium on the dependent variables showed strong evidence supporting H_1_ for processing time (BF_10_ = 21.306) and moderate evidence supporting H_0_ for response length (BF_10_ = 0.201), integrated understanding (BF_10_ = 0.169), and calibration (BF_10_ = 0.173). With respect to an effect of context, there was moderate evidence supporting H_0_ for all dependent variables: processing time (BF_10_ = 0.178), response length (BF_10_ = 0.183), integrated understanding (BF_10_ = 0.148), and calibration of comprehension performance (BF_10_ = 0.144). Overall, these results confirmed the equivalence of performance across conditions for response length, integrated understanding, and calibration, as well as the difference across conditions in processing time.

## Discussion

This study uniquely investigated the potential impact of medium on processes and outcomes of multiple source use. At the same time, it examined whether the informational context might modify any effects of medium on learners’ processing and integration of information, as well as on their calibration of comprehension performance. For each of our research questions, we formulated alternative hypotheses grounded in theoretical assumptions and mixed findings from a few prior studies. Our alternative hypotheses about the lack of any effects of medium and context were confirmed, except for processing time. For this aspect of students’ engagement with the learning materials, results showed a main effect of medium in favor of videos, which were processed for a longer time than were texts. If any medium effect would emerge, we had expected it to be in the opposite direction based on the study by List and Ballenger (2019). Those authors found that when the first source was a text, it was processed for a longer time than was a video, whereas no difference with respect to processing time was found for the second source. However, List and Ballenger did not use an Internet video (Delgado et al. 2021; Salmerón et al., 2020), like we did in this study, and only the first source they presented was expository (the second had a more narrative tone). One possible interpretation of our finding regarding processing time is that students perceived the visual materials as more enjoyable, interesting, and beneficial for learning than the textual materials (Wilson et al., 2018). This seems consistent with the great popularity of videos among young people, as well as with the fact that our participants reportedly preferred learning from videos over learning from texts (see section on Participants). Nevertheless, longer processing did not result in better integration of information across sources or better calibration, which indicates that longer processing of the videos was neither effective nor efficient.

Increasingly, digital texts and videos seem to take center stage in educational contexts (Baron, 2021; Baron & Mangen, 2021; Burin et al., 2021). This digital shift has caused some concern among both educators and researchers because research has indicated that printed texts are advantageous in terms of comprehension performance, as compared with digital texts (Clinton, 2019; Delgado et al., 2018). This suggests that traditional, paper based reading can prime a deeper processing of information than does digital reading, even among postsecondary students, which is consistent with the shallowing hypothesis by Annisette and Lafreniere (2017). According to our findings, further shallowing does not seem to occur when moving from digital texts to videos as learning materials, however. As a consequence, similar comprehension and metacomprehension can be observed between these two mediums. This may be due to the largely parallel rise of digital texts and videos as learning tools, with none of them gaining precedence as the deeper, more reflective thought technology. Of note is also that consistent results were reported by List (2018), who did not find any differences with respect to comprehension and integration between digital texts and videos. Our additional finding that these mediums were similar with respect to metacomprehension (i.e., calibration) seems consistent with the equivalence between them with respect to comprehension. While our results may be interpreted as a positive message for teachers and faculty currently presenting or assigning digital learning materials, they, of course, do not speak to potential differences between learning from digital texts and videos on the one hand and printed texts on the other.

Regarding the impact of context, we again found support for the null hypotheses in that there were no effects of the authoritativeness of the informational context on any of the process or outcome variables. Nor were any interactions between medium and context found. These findings can be interpreted in light of the context model included in the RESOLV framework proposed by Rouet, Britt, et al. (2017; see also Britt et al., 2018). If students do not discriminate between a discussion among friends and a classroom discussion, between a friend and a professor, and/or between Facebook and Moodle, their context model representation will also not include any information about authoritativeness. Young people rely heavily on social media, such as Facebook, for information. However, Facebook users may easily spread inaccurate information on their social medium profiles and encourage others to use such information in making decisions (Di Domenico et al., 2021). Facebook also includes accurate information from highly reliable sources, of course, which makes it essential to discriminate between various social media sources. Still, an appropriate context model does not seem to be formed if students consider a context in which a friend posts information on Facebook after a discussion with other friends as authoritative as a context in which a professor posts information on Moodle after a classroom discussion. As a consequence, equivalence between these informational contexts with respect to processes and outcomes is a likely result. That the students did not seem to distinguish between the authoritativeness of the two informational contexts may be a matter of concern.

Given the wide spread of fake news and misinformation in present-day societies, it is crucial to help students in developing the abilities and habits of paying attention to, discerning, and selecting authoritative informational contexts. Indeed, if consumers of information do not take such authoritativeness into account, they may easily fall prey to people interested in disseminating misinformation with potentially dangerous, even life-threatening effects.

### Limitations and directions for future research

As any study, the current one comes with several limitations. First, the topic of the learning materials ‒ social media ‒ was likely quite familiar to the participants and the text may not have represented enough of a challenge to them. Presumably, a less familiar and more complex topic, requiring deeper comprehension of explanatory mechanisms, could have led to different effects on processing and outcome variables. Future research should therefore explore the role of topic complexity when simultaneously considering the roles of medium and informational context.

Second, the type of videos should also be taken into account. We used an Internet-type video showing a person speaking in front of the camera, such as in many YouTube videos viewed on the Web. As such, there were no static or dynamic pictures, nor any seductive details in the videos that could cause cognitive overload or distractions and lead to mind wandering. Rather, our videos were merely the spoken versions of the two texts, which might have made them too similar to the texts in terms of the demands on comprehension and metacomprehension. Multimodal videos could therefore be used instead of Internet videos in future studies.

Third, the two informational contexts might not have been sufficiently distinct in terms of authoritativeness. We used two platforms, Facebook and Moodle, on which the learning materials were posted by a friend and a professor, after a discussion with other friends out of school or a classroom discussion with a professor, respectively. In doing this, we kept the research design as rigorous as possible to investigate the role of the informational context. Specifically, we held the content of the two learning materials and the source information constant across contexts. However, rigor, cleanliness, and quite subtle manipulations of the two independent variables, as described, came at the cost of external validity and, even more importantly, might have minimized the chances of revealing effects on the outcome variables. Moreover, we did not check whether the participants perceived the Moodle platform as more authoritative than Facebook. Undoubtedly, more research is needed to shed further light on the roles of medium and informational context when students read digital texts and watch videos to learn about various issues. More complex designs aiming for higher external validity and, thus, more generalizable findings, should not only manipulate information content and source information but also include other features that characterize digital texts and videos. Manipulation checks will also be important to ensure that participants actually perceive a context as more authoritative than another.

Fourth, we took into account the issue of authenticity when manipulating our context variable as the complementary texts/videos were embedded in the two online platforms, that is Facebook and Moodle, in an authentic way (Figs. 1 and 2). However, we cannot maintain that the study occurred in a naturalistic situation where learners were involved in a real classroom discussion about the topic of social media. In different areas of learning research students are often asked “to imagine” a given situation to manipulate experimental conditions (e.g., Latini et al., 2019; McCrudden et al., 2010). Even if we considered authenticity when embedding the learning materials in the two platforms, an even more naturalistic manipulation requires that students really perform the activities that lead to knowing more on a topic. Next investigations need to be featured by a higher degree of authenticity in all the aspects of a learning setting.

Fifth, we considered a number of relevant control variables but did not take participants’ topic-specific beliefs into account. The two sources presented complementary rather than contrasting points of view. Still, students’ prior beliefs about social media might have come into play and influenced their processing, comprehension, and calibration of comprehension performance. In future studies in this area, the individual difference variable of topic-specific beliefs should therefore be included as a control variable.

## Conclusions

Notwithstanding these limitations, the current study is not a trivial step in educational research on the potential effects of mediums and contexts. It suggests that digital medium, text or video, does not make a difference in terms of comprehension and integration of information from multiple complementary sources. The relevance of this finding is obvious given the massive use of such learning materials among students (Baron, 2021), not least during the last years due to the online shift in learning driven by the COVID-19 pandemic. The study also suggests that students may not take differences between online informational contexts that vary in terms of authoritativeness into account when working with digital learning materials. This highlights the importance of increasing students’ awareness of the need to consider contextual cues regarding expertise and reliability when searching for information, either in texts or videos, to know more about an issue.

## Footnote

^1^The results for processing time did not change when the ANCOVA was performed with the total sample of 255 participants. There was an effect of medium, *F*(2, 247) = 4.45, *p* = .012, η^*2*^_*p*_ = 0.035, while neither the main effect of context, *F*(1, 247) = 0.45, *p* = .503, η^*2*^_*p*_ = 0.002, nor the interaction, *F*(2, 247) = 1.52, *p* = .219, η^*2*^_*p*_ = 0.012, was significant. The covariate of task value of learning from videos uniquely adjusted processing time, *F*(1, 247) = 5.04, *p* = .026, η^*2*^_*p*_ = 0.020, while the effect of the other covariate, reading comprehension, was only marginal, *F*(1, 247) = 3.80, *p* = .052, η^*2*^_*p*_ = 0.012.

## Electronic Supplementary Material

Below is the link to the electronic supplementary material.

Supplementary Material 1
