# Supplementary material for: Do medium and Context Matter when learning from multiple complementary Digital texts and videos?
Source: Instr Sci. 2022 Sep 11;50(5):653–79. doi: 10.1007/s11251-022-09591-8 (PMC9464432; doi:10.1007/s11251-022-09591-8)
Supplement: Supplementary file 1 — Supplementary Material 1 [file 11251_2022_9591_MOESM1_ESM.docx]

**Supplementary Material**

**The Two Learning Materials (English version of the written/spoken texts)**

From Latini, N., Bråten, Anmarkrud, Ø., & Salmerón, L. (2019, pp. 11-12). Investigating effects of reading medium and reading purpose on behavioral engagement and textual integration in a multiple text context. *Contemporary Educational Psychology, 59*. Article e101797. <https://doi.org/10.1016/j.cedpsych.2019.101797>

**Social Media – Friend or Foe?**

Social media are places where you can get in touch with other people. Especially for young people, social media have become an almost indispensable part of life. Today, practically everyone uses digital devices such as smartphones, smart watches, tablets, or laptops. Therefore, social media follow us everywhere and around-the-clock. But is this only a good thing? What does being social on social media really mean? And does this mean the same for everybody? In this article, we will among other things see that the use of social media does not necessarily make us happier or wiser.

Many have expressed concerns that the increasing use of social media will actually lead to more people becoming isolated. Such a concern seems justified because it has been shown that people can feel more socially isolated the more time they spend on social media. It is a paradox that those who use social media more than two hours a day have twice as high risk of feeling lonely than those who use less than half an hour. Those who use social media relatively often respond “yes” to questions such as “I feel that people hardly know me,” or “I feel people are around me, but not together with me”. However, it is somewhat unclear whether those who report much use of social media feel isolated because they are often on social media, or whether they use social media because they feel isolated.

There are clear indications that the relationship with friends on social media may differ from that with friends in real life. A close and intimate friendship depends on a mutual relationship and that people are there for each other. However, the question is whether this can be expected of contacts on social media to the same extent. Loyalty and trust between people who know each other well in the real world are probably different than what occurs on social media. When Facebook users were asked to ask their contacts on Facebook to do them a favor, only ten percent of the contacts were willing to give 10 minutes of their time. By comparison, most of the friends they spent time with on a daily basis offered to help, which may indicate that one’s contacts on social media are not necessarily the same as close friends.

However, men and women tend to use social media differently. Many men do not primarily use social media to stay in touch with friends and family. Instead of using social media to communicate with persons who are close to them, they use them to communicate with people they do not know well or with strangers. This may involve that men use social media to discuss and comment on controversial societal and political issues. The social aspect is not the focus of such discussions, but rather the discussion itself, which is regarded as a form of public communication they figure may be rendered in other contexts. Men who primarily use social media in such a way may feel that they do not belong to any social community, even though they are very active on social media.

Not only gender differences are relevant in this connection, however.

Young people are different in regard to education and cultural preferences, which appear to be reflected in their use of social media. One example of this is the use of Twitter. Researchers have analyzed the hash-tags of a large group of Twitter users and compared these with the users’ educational level. People with lower education seemed to be very occupied with celebrities and entertainment and produced little content themselves. Instead, they often liked postings from others, usually celebrities, and reposted these on their own Twitter accounts without any critical evaluation of the content. It has also been shown that Facebook users with lower education spend much time on their own profile, and on posting private photos and personal information. This may give the impression that they are relatively unconcerned about information security and personal privacy and uncritical as to what they share on social media.

Social media are here to stay. They play an increasingly greater role in a digital world. The use of social media affects both ourselves and the relationships we have with others. Perhaps social media in themselves are neither friend nor foe, but what we ourselves make of them?

**Social Media = Social People?**

Humans are social beings. The group is important. Through the social we also experience ourselves. But what happens when the social meeting places are moved from the real reality to the digital reality? Because social media are now used by all groups of people, particularly by young people, it is important to understand how social media can intervene in people's lives. Does more use of social media necessarily lead to more social activity and a closer connection to the group, or can they just as easily create passivity and distance? In this article, we will see, among other things, that the use of social media is significant for how we establish and maintain relationships in digital networks.

It is undoubtedly the case that social media can contribute to expanding people's networks. This is because it is a simple and effective form of contact that practically has exploded globally with the emergence of digital technologies. Before social media became particularly prevalent, young adults usually had between 10 and 15 friends, while today they have far more contacts on social media than they have friends with whom they spend time. Social media make it easier to establish contact with many people independently of physical distance. Profiles on different social media provide information on shared interests and shared contacts, thus making it easier to acquire a wide circle of acquaintances. Through this circle of acquaintances, one can get continuous updates on different happenings and events. Such updates can give a social media user access to new acquaintances such that the network constantly grows through a kind of snowball effect.

Social media allow for insight into other people's lives in a way that was not previously possible. But on social media many people want to present themselves favorably, for example by posting photos from parties and generally portraying themselves as happy, attractive and popular. When people consciously present themselves in a positive way, social comparisons based on such a staged “reality” will be different from social comparisons one makes in real life. This is because they primarily involve an upward social comparison with people who appear socially successful. Those who follow such updates may thus be comparing themselves with unrealistic idealized images of others’ lives. Because young people are in a period of life where they are especially concerned with comparing themselves to others, frequent use of social media will give them many opportunities to make such comparisons.

However, gender differences have been shown in regard to the use of social media. For women, the social is in focus and they spend much time on contact with friends and family. This may have the character of “everyday socializing”, that is using social media to keep up to date with friends, comment on their photos and postings, and post updates related to one’s own everyday life. The emphasis on communication with private contacts is reflected in the way women discuss on social media. This means that when they discuss on social media, they prefer to discuss with people they know well. That is to say that women to a large degree consider discussions on social media private communication with their contacts, and not public communication that they must assume will be rendered in other contexts. Women who are not on social media may thus experience that they are not participating in an important social arena.

Young people’s relationship to social media can also be influenced by social and cultural differences between the users. It may seem that people with higher education often use social media to acquire and share knowledge. For example, this may involve that they use Twitter to read and write about topics such as politics, training, and health, or that they use Facebook to share their own cultural experiences and current political and social issues. Well-educated people are also able to assess the reliability of the information they access on social media, which is a prerequisite for making well-informed choices. On the other hand, people with higher education relatively seldom post photos of themselves or their family on social media. This may be related to the fact that people with higher education are concerned about information security and personal privacy, and thus are cautious about what they share.

Social media do not appear to change people’s fundamental need for a sense of belonging. People still need other people. Presumably this is part of the driving force behind the prevalence of social media. The question is whether social media can satisfy people’s social needs in the same way as real life.

Table 1S

Coding System for Scoring the Written Responses to the Four Integrative Questions (Based on Latini et al., 2019)

Score Definition Example

**Question 1: Can people’s presentation of themselves in social media make a difference to the mental health of the users of social media?**

| 0  1  2  3 | Participants gave no response or described irrelevant information  Participants described an issue as discussed in one of the texts/videos  Participants described an issue as discussed in both texts/videos without integrating information about that issue across the texts/videos  Participants described an issue as discussed in both texts/videos as well as integrated information about that issue across the texts/videos | Yes, unfortunately, this can happen very often.  Yes, because, especially adolescents tend to make comparisons between their own lives and the lives that people share.  Yes sure. As it is well expressed in the text, social media can cause isolation and passivity for those who use it frequently, as they tend to think they do not have many friends in real life. The video refers to how social media can be a reason to feel inferior: people are subjected to a series of unrealistic images, the comparison with which generates inferiority complex.  Yes, sometimes people show their life on social networks differently from what they really are, they create posts in which they show countless meetings with friends or posts in which they always seem happy, but this can lead themselves and even those who watch them to feel more alone. |
| --- | --- | --- |

**Question 2: Is there any connection between education and the use of social media?**

| 0  1  2  3 | Participants gave no response or described irrelevant information  Participants described an issue as discussed in one of the texts/videos  Participants described an issue as discussed in both texts/videos without integrating information about that issue across the texts/videos  Participants described an issue as discussed in both texts/videos as well as integrated information about that issue across the texts/videos | I think there is a relationship because nowadays we are all immersed in social media.  Yes, poorly educated people on Facebook spend a lot of time on their profile for example those with a lower level of education show a certain degree of passivity and lack of critical sense (Twitter), or poor conception of privacy (Facebook).  Yes, in fact in the videos differences in the use of social media are presented between those with a high level and those with a low level of education. The former use them to share or acquire knowledge, the latter mainly to comment or share posts written by important people.  Yes, it has been observed that people with a higher level of education tend to use social networks to acquire knowledge, whereas people with a lower level of education tend to use them for entertainment (for example following a famous person). |
| --- | --- | --- |

**Question 3: Can the use of social media affect friendships in any way?**

| 0  1  2  3 | Participants gave no response or described irrelevant information  Participants described an issue as discussed in one of the texts/videos  Participants described an issue as discussed in both texts/videos without integrating information about that issue across the texts/videos  Participants described an issue as discussed in both texts/videos as well as integrated information about that issue across the texts/videos | The use of social media can affect friendships.  Yes, when you need help, Facebook friends are rarely ready to give you time.  Before social media spread, young people had between ten and fifteen friends while today they have more “friends” on social media with whom they spend time. The relationships that are established between friends on social networks are very different from what can be created in reality, especially concerning loyalty and trust.  Social media undoubtedly have the ability to greatly expand the network of social contacts, however the knowledge acquired in this way has a different quality compared with personal relationships with close people. |
| --- | --- | --- |

**Question 4: Is there any connection between gender and the use of social media?**

| 0  1  2  3 | Participants gave no response or described irrelevant information  Participants described an issue as discussed in one of the texts/videos  Participants described an issue as discussed in both texts/videos without integrating information about that issue across the texts/videos  Participants described an issue as discussed in both texts/videos as well as integrated information about that issue across the texts/videos | Yes, the use of social media has created and creates social differences.  Yes. Men, for example, seem to spend more time discussing politics on social media than women.  Yes. Men prefer to get in touch with people they don’t know well or don’t know at all, women spend more time in contact with friends and family.  It would seem that men and women differ for the content shared: women share more aspects of their private life whereas men use social networks more to meet new people. |
| --- | --- | --- |

Table 2S

Means and (Standard Deviations) for all Covariates by Condition (*N* = 255)

|  | Unauthoritative context (Facebook) |  |  | Authoritative context  (Moodle) |  |  |
| --- | --- | --- | --- | --- | --- | --- |
|  | Texts  (*n* = 43) | Videos  (*n* = 43) | Mixed  (*n* = 44) | Texts  (*n* = 42) | Videos  (*n* = 39) | Mixed  (*n* = 44) |
| Perceived prior knowledge | 38.67  (10.97) | 38.79  (11.01) | 40.64  (9.12) | 42.07  (11.43) | 37.46  (10.39) | 41.93  (9.12) |
| Reading comprehension | 10.19  (2.40) | 10.47  (1.96) | 9.68  (2.74) | 10.52  (2.38) | 9.87  (2.74) | 10.55  (2.64) |
| Cognitive reflection | 3.79  (1.87) | 3.53  (1.93) | 3.18  (2.11) | 3.64  (1.77) | 3.69  (1.79) | 3.05  (1.86) |
| Task value for learning from text | 93.83  (10.23) | 92.13  (10.67) | 88.61  (11.91) | 91.59  (11.84) | 87.35  (10.83) | 89.13  (11.87) |
| Task value for learning from videos | 89.88  (11.56) | 89.32  (10.83) | 85.97  (11.10) | 89.21  (9.69) | 86.48  (11.37) | 88.90  (10.73) |

*Note*. Univariate tests from a multivariate analysis of variance showed no statistically significant differences across subgroups, with *F*(5, 249) = 1.48, *p* = .195, η*^2^_p_* = .029, for perceived prior knowledge; *F*(5, 249) = .86, *p* = .507, η*^2^_p_* = .017, for reading comprehension; *F*(5, 249) = 1.01, *p* = .414, η*^2^_p_* = .020, for cognitive reflection; *F*(5, 249) = 1.97, *p* = .083, η*^2^_p_* = .038, for task value of learning from text; and *F*(5, 249) = 1.12, *p* = .347, η*^2^_p_* = .022, for task value of learning from videos.
